# Recent Advances in the Use of *Ganoderma lucidum* and *Coriolus versicolor* Mushrooms to Enhance the Anticancer Efficacy of EGFR-Targeted Drugs in Lung Cancer

**DOI:** 10.3390/pharmaceutics17070917

**Published:** 2025-07-15

**Authors:** Hang Zhang, Longling Wang, Yuet Wa Chan, William C. Cho, Zhong Zuo, Kenneth K. W. To

**Affiliations:** 1 School of Pharmacy, Faculty of Medicine, The Chinese University of Hong Kong, Hong Kong SAR, China; zhariel@link.cuhk.edu.hk (H.Z.); ottly@link.cuhk.edu.hk (L.W.); joanzuo@cuhk.edu.hk (Z.Z.); 2 Institute of Chinese Medicine, The Chinese University of Hong Kong, Hong Kong SAR, China; judychanyw@cuhk.edu.hk; 3 Department of Clinical Oncology, Queen Elizabeth Hospital, Hong Kong SAR, China; williamcscho@gmail.com

**Keywords:** EGFR-TKI, Lingzhi, Yunzhi, NSCLC, drug resistance

## Abstract

Lung cancer is the leading cause of cancer-related deaths worldwide. Non-small cell lung cancer (NSCLC) is the major subtype, accounting for more than 85% of all lung cancer cases. Recent advances in precision oncology have allowed NSCLC patients bearing specific oncogenic epidermal growth factor receptor (EGFR) mutations to respond well to EGFR tyrosine kinase inhibitors (TKIs). Due to the high EGFR mutation frequency (up to more than 50%) observed particularly in Asian NSCLC patients, EGFR-TKIs have produced unprecedented clinical responses. Depending on their binding interactions with EGFRs, EGFR-TKIs are classified as reversible (first-generation: gefitinib and erlotinib) or irreversible inhibitors (second-generation: afatinib and dacomitinib; third-generation: osimertinib). While the discovery of osimertinib represents a breakthrough in the treatment of NSCLC, most patients eventually relapse and develop drug resistance. Novel strategies to overcome osimertinib resistance are urgently needed. In Asian countries, the concomitant use of Western medicine and traditional Chinese medicine (TCM) is very common. *Ganoderma lucidum* (Lingzhi) and *Coriolus versicolor* (Yunzhi) are popular TCMs that are widely consumed by cancer patients to enhance anticancer efficacy and alleviate the side effects associated with cancer therapy. The bioactive polysaccharides and triterpenes in these medicinal mushrooms are believed to contribute to their anticancer and immunomodulating effects. This review presents the latest update on the beneficial combination of Lingzhi/Yunzhi and EGFR-TKIs to overcome drug resistance. The effects of Lingzhi/Yunzhi on various oncogenic signaling pathways and anticancer immunity, as well as their potential to overcome EGFR-TKI resistance, are highlighted. The potential risk of herb–drug interactions could become critical when cancer patients take Lingzhi/Yunzhi as adjuvants during cancer therapy. The involvement of drug transporters and cytochrome P450 enzymes in these herb–drug interactions is summarized. Finally, we also discuss the opportunities and future prospects regarding the combined use of Lingzhi/Yunzhi and EGFR-TKIs in cancer patients.

## 1. Introduction

EGFR-mutated NSCLC represents a clinically significant subset of lung cancers, characterized by oncogenic driver mutations that promote tumor growth, survival, and progression. While EGFR-TKIs such as gefitinib, erlotinib, and osimertinib have revolutionized the treatment of EGFR-mutated NSCLC by targeting these mutations, therapeutic resistance remains a major challenge. Mechanisms of resistance, including secondary mutations (e.g., T790M and C797S), bypass pathway activation (e.g., *MET* amplification), and phenotypic transformations (e.g., epithelial–mesenchymal transition (EMT) or small cell lung cancer (SCLC) transformation), often limit the long-term efficacy of EGFR-TKIs [1,2,3,4]. Furthermore, the tumor microenvironment (TME) of EGFR-mutated NSCLC, characterized by a low tumor mutation burden (TMB), impaired T cell activity, and high levels of immunosuppressive cytokines, further complicates treatment outcomes [5,6].

*Ganoderma lucidum* (Lingzhi) and *Coriolus versicolor* (Yunzhi), two medicinal mushrooms widely used in traditional Chinese medicine (TCM) in Asian countries, have emerged as promising complementary therapies in cancer treatment. Their bioactive compounds, including polysaccharides and triterpenoids, exhibit a broad range of antitumor activities, such as the inhibition of oncogenic signaling pathways, suppression of angiogenesis, reversal of multidrug resistance (MDR), and modulation of tumor immunity [7,8]. They enhance immune function by activating natural killer (NK) cells, T cells, and macrophages while reducing immunosuppressive cells such as regulatory T cells (Tregs) and myeloid-derived suppressor cells (MDSCs) [9,10,11,12]. Lingzhi and Yunzhi are also known to enhance the efficacy of conventional cancer therapies, including chemotherapy, radiotherapy, and targeted therapies, by inhibiting various oncogenic signaling pathways and reversing MDR [13,14,15]. The two medicinal mushrooms are also known to reduce the immunosuppressive and oxidative stress effects caused by chemotherapeutic drugs while enhancing their efficacy [10]. Yunzhi has also been demonstrated to reduce fatigue and enhance immune recovery in cancer patients [16]. Moreover, both mushrooms alleviate treatment-related side effects, such as fatigue, nausea, and immunosuppression, thus improving the quality of life of cancer patients [10,17,18,19]. Recent studies suggest that Lingzhi/Yunzhi may augment the efficacy of EGFR-TKIs by targeting EGFR-associated resistance mechanisms, reducing immunosuppression in the TME, and enhancing immune responses [15,20,21,22].

This review summarizes the latest advances in the beneficial therapeutic effects of Lingzhi/Yunzhi in overcoming drug resistance, strengthening immunity, and improving clinical outcomes in EGFR-mutated NSCLC. These medicinal mushrooms may be used as adjuvant therapies to address the current challenges in the management of EGFR-mutated NSCLC [23].

## 2. Drug Resistance to EGFR-TKIs

### 2.1. Resistance Mechanisms to First- and Second-Generation EGFR-TKIs

The resistance mechanisms of first- and second-generation EGFR-TKIs can be categorized into three main types: EGFR-dependent changes, activation of bypass or downstream pathways, and phenotypic transformations. The most prominent mechanism is the EGFR T790M mutation, which accounts for 50–60% of resistance cases against the first- and second-generation competitive EGFR-TKIs. This mutation leads to drug resistance by increasing the affinity of the EGFR kinase for ATP. Other EGFR mutations, such as L747S and T854A, contribute to resistance but are less common. The activation of bypass pathways, including *HER2* amplification, *MET* amplification, and PIK3CA/KRAS mutations, promotes tumor survival and proliferation. Moreover, phenotypic transformations, such as EMT or transformation to SCLC, further drive resistance [2,3,4].

### 2.2. Drug Resistance to Osimertinib in First-Line and Second-Line Treatment

**Figure 1:**
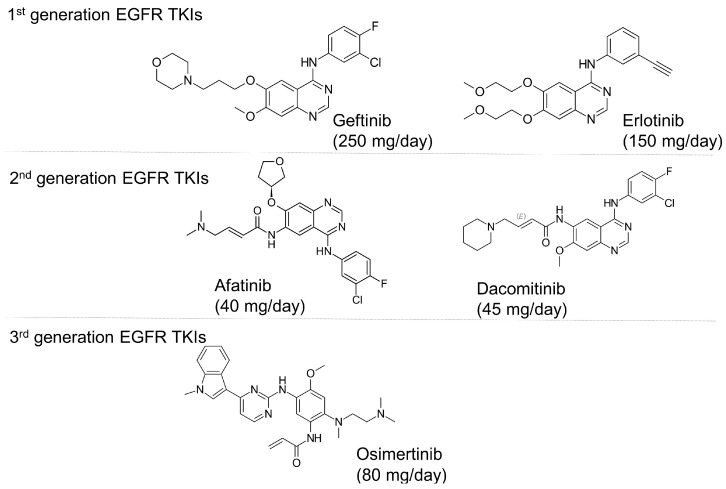
Chemical structures and recommended daily dosage of the US Food and Drug Administration (FDA)-approved EGFR TKIs.

Osimertinib is the only third-generation EGFR-TKI clinically approved by major regulatory authorities for the treatment of EGFR T790M-positive patients who have progressed following treatment with first- or second-generation EGFR-TKIs. Osimertinib was also approved in 2018 as a first-line therapy for advanced EGFR-mutated NSCLC, regardless of T790M mutation status. Figure 1 shows the chemical structures of US Food and Drug Administration (FDA)-approved EGFR TKIs. While the discovery of osimertinib represents a breakthrough in the treatment of NSCLC, most patients eventually relapse and develop resistance to treatment. Osimertinib resistance arises from EGFR-dependent mechanisms (including tertiary mutations, such as C797S, and *EGFR* amplification) or EGFR-independent mechanisms (such as *MET*/*HER2* amplification, KRAS/BRAF mutations, and oncogenic fusions) [1]. Histological transformations (e.g., SCLC) and EMT also contribute, subsequently activating bypass pathways (MAPK, PI3K-Akt) and promoting tumor survival and progression [1,3].

#### 2.2.1. EGFR-Dependent Resistance Mechanisms [24]

Tertiary EGFR mutations, particularly C797S (10–26% in second-line; 7% in first-line settings), disrupt osimertinib binding and confer resistance, with treatment effectiveness depending on its allelic context (*cis* versus *trans* with T790M). Rare EGFR mutations like L792H, L718Q, G796R, and G724S also hinder osimertinib binding, while L718Q retains sensitivity to earlier-generation TKIs when T790M is absent. *EGFR* amplification, observed mainly in second-line settings, enhances EGFR signaling and bypasses osimertinib inhibition. Additionally, T790M dynamics show that its loss during progression is linked to alternative resistance mechanisms (e.g., *MET* amplification, KRAS mutations) and poorer outcomes.

#### 2.2.2. EGFR-Independent Resistance Mechanisms

Bypass pathway activation is a pivotal osimertinib resistance mechanism that involves the emergence of alternative signaling pathways or histological transformations to drive cancer growth [25]. Among these mechanisms, *MET* amplification represents the most commonly reported one, occurring in 15% of first-line and 19% of second-line cases. This could co-occur with EGFR mutations such as C797S, thereby stimulating downstream pathways (MAPK, PI3K-Akt). *HER2* amplification (2–5%) and RAS–MAPK pathway mutations (e.g., KRAS G12D, BRAF V600E) further drive resistance, while PI3K pathway activation via PIK3CA mutations or PTEN loss promotes tumor survival. Rare oncogenic fusions (e.g., BRAF, RET, ALK) are found in 3–10% of cases and can co-occur with EGFR mutations. Histological transformation to SCLC, associated with TP53 and RB1 inactivation, occurs in 4–15% of cases. Additionally, EMT, characterized by reduced E-cadherin and increased vimentin, contributes to resistance through transcription factors such as ZEB1 and TWIST1.

## 3. Potentiation of Antitumor Efficacy of EGFR-TKIs by Medicinal Mushrooms

### 3.1. Lingzhi Inhibits Various Oncogenic Pathways and Overcomes Drug Resistance in EGFR-Mutated NSCLC

Lingzhi demonstrates potent antitumor effects in EGFR-mutated NSCLC by downregulating EGFR expression, inhibiting key oncogenic pathways (PI3K/AKT/mTOR, ERK, Wnt/β-catenin), and suppressing EMT and angiogenesis. It addresses resistance to EGFR-TKIs by targeting bypass mechanisms, reactivating p53, and reversing MDR. Moreover, Lingzhi has also been reported to inhibit telomerase activity, thus limiting cancer cell immortality [14,15,26]. The multifaceted biological activities of Lingzhi in EGFR-mutated NSCLC are described below.

#### 3.1.1. Inhibition of EGFR and Its Downstream Signaling Pathways

**Figure 2:**
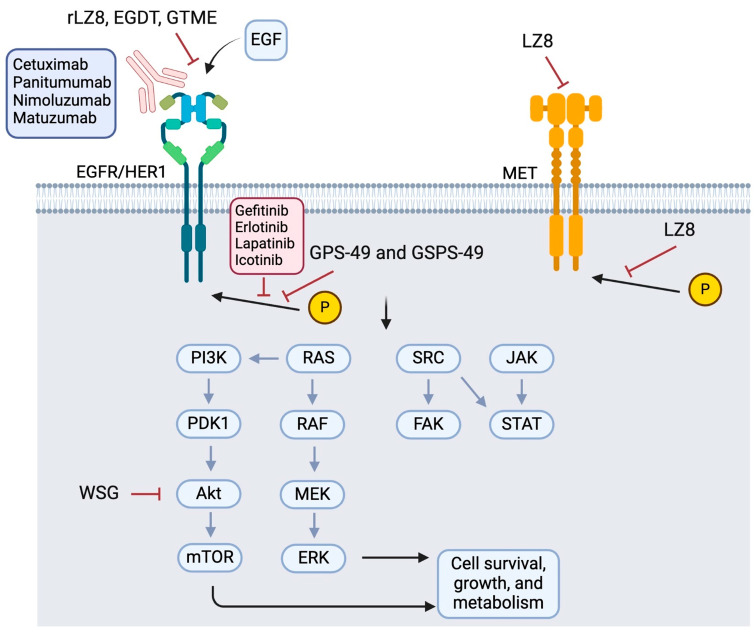
Reported molecular targets of Lingzhi in the EGFR signaling pathway. Abbreviations: rLZ8: recombinant Ling Zhi-8 (a protein isolated from *Ganoderma lucidum*); EGDT: Ergosta-7,22-diene-2β,3α,9α-triol (a sterol); GTME: *G. tsugae* methanol extract; GPS-49 and GSPS-49: *Ganoderma lucidum* polysaccharides or sulfated polysaccharides isolated on day 49; WSG: a water-soluble glucose-enriched polysaccharide from *Ganoderma lucidum*; LZ-8: a medicinal peptide of Lingzhi.

Numerous active components have been identified from Lingzhi that can inhibit EGFR pathways to suppress tumor growth, induce apoptosis, and block angiogenesis (Figure 2 and Table 1). WSG, a water-soluble polysaccharide isolated from Lingzhi with an average molecular mass of approximately 1000 kDa, was shown to significantly inhibit lung tumor growth, reduce the size of metastatic nodules in the lungs, and prolong the survival of LLC1-bearing mice [7]. Mechanistically, WSG was found to inhibit the phosphorylation of ERK1/2 in NSCLC cells upon either EGF or TGFβ stimulation. It also inhibited the phosphorylation of several other downstream signaling molecules, including FAK, AKT, and Smad2. Furthermore, WSG was reported to induce the degradation of TGFβ and EGF receptors via the proteasomal and lysosomal pathways, respectively [7]. In tongue cancer cells, WSG was shown to dramatically reduce cell viability and colony formation, increase sub-G1 and G2/M populations, and induce apoptotic responses [27]. The enhanced apoptosis was associated with an increase in the Bax/Bcl2 ratio and reduction in EGFR/AKT phosphorylation. Furthermore, the WSG + cisplatin combination was found to produce synergistic anticancer effects [27].

rLZ-8 (Ling Zhi-8), an immunoregulatory protein extracted from Lingzhi [33], regulates multiple immune cells, including T cells. It was shown to induce cell cycle arrest and apoptosis by downregulating the expression of wild-type and mutated EGFR and inhibiting EGFR downstream effectors (AKT and ERK1/2) in lung cancer cells in vitro [15]. In a lung tumor-bearing mouse model, rLZ-8 was also shown to effectively inhibit cancer progression and reduce tumoral EGFR expression in vivo. Mechanistically, rLZ-8 was found to decrease the plasma membrane expression of EGFR by altering EGFR localization and to enhance the EGF-induced degradation of EGFR [15]. rLZ-8 could bind to EGFR to induce EGFR autophosphorylation at tyrosine-1045 and trigger ubiquitination by inducing the formation of EGFR/Cbl complexes, subsequently leading to EGFR protein degradation. This working model was further supported by the fact that Cbl-shRNA abolished rLZ-8-induced EGFR degradation [15]. In other human cancer types besides NSCLC, rLZ-8 (0.2–2 mg/mL) was shown to significantly inhibit cancer cell proliferation including breast (MDA-MB-468), hepatocellular carcinoma (HCC) (Hep3B), and melanoma (B16F10). It also exhibited the dose-dependent inhibition (4–8 mg/kg) of tumor xenograft growth in an orthotopic HCC NOG-mouse model [28]. In the HCC cell line Hep3B, rLZ-8 was shown to specifically bind to EGFR and readily penetrate the cell membrane, subsequently disrupting endosomal recycling and inducing apoptosis. Interestingly, the antitumor efficacy of rLZ-8 on five patient-derived tumor xenograft (PDX) models of HCC (LI6280, LI1097, LI0050, LI0334, LI6611) was directly correlated with tumoral EGFR expression levels, suggesting the mechanistic involvement of EGFR. Molecular docking and protein cross-linking studies revealed that rLZ-8 directly blocks the EGFR dimerization interface via an epitope (S222/K269) on the extracellular domain of EGFR [28]. The formation of functional EGFR dimers and downstream EGFR signaling was therefore inhibited by rLZ-8 [28].

Ergosta-7-22-diene-2β,3α,9α-triol (EGDT), a sterol isolated from the mycelia of Lingzhi, demonstrated significant concentration-dependent (10–50 μM) anti-proliferative effects on nasopharyngeal carcinoma (NPC) cells, induced G0/G1 phase cell cycle arrest, and promoted apoptosis in vitro. EGDT was shown to downregulate EGFR at both mRNA and protein levels, which was associated with the significant inhibition of EGFR downstream RAF/MEK/ERK and PI3K/AKT signaling pathways. Additionally, EGDT (10 mg/kg) exhibited robust antitumor activity in NPC xenograft models in immunocompromised mice [29].

The methanol extract of *Ganoderma tsugae* (GTME), a polypore mushroom closely related to Lingzhi, was also reported to inhibit the proliferation of an EGFR-high-expressing epidermoid carcinoma cell line (A-431) and capillary-like tube formation in human umbilical vein endothelial cells (HUVECs). GTME (50–200 μg/mL) inhibited EGFR and VEGF expression, thereby reducing VEGF secretion from A-431 cells [30]. Since VEGF plays a pivotal role in angiogenesis, tumor growth, and metastasis, GTME may be useful for antitumor treatment by inhibiting tumor vasculature.

Lingzhi contains numerous biologically active components, among which polysaccharides (GPS) and their sulfated form (GSPS) are known to possess potent antitumor, immunomodulatory, anti-inflammatory, and neuroprotective effects. GPS is produced from the fruiting bodies, spores, or mycelia of Lingzhi. Accumulating evidence suggests that GPS is more abundant in the mycelia than in the fruiting bodies and spores. Therefore, commercially available GPS is usually obtained from Lingzhi mycelia. During industrial production, different supplements can be added to the culture medium of Lingzhi mycelia to increase the yield of mycelial biomass and derived GPS. Interestingly, the physicochemical characteristics and antitumor activities of GPS/GSPS from Lingzhi mycelium have been shown to vary with the harvest time of the liquid Lingzhi mycelial culture [32]. The optimal time to harvest GPS and GSPS was found to be 49 days from the mycelial culture. GPS and GSPS (100–300 μg/mL), isolated on day 49, demonstrated significant anticancer effects without impacting normal fibroblasts. The inhibitory effect of GPS-49 and GSPS-49 on the signaling networks mediated by EGFR and the transforming growth factor beta receptor (TGFβR) was found to be the most optimal [32].

#### 3.1.2. Modulation of VEGF-Dependent Angiogenesis Program by Lingzhi/Yunzhi

**Figure 3:**
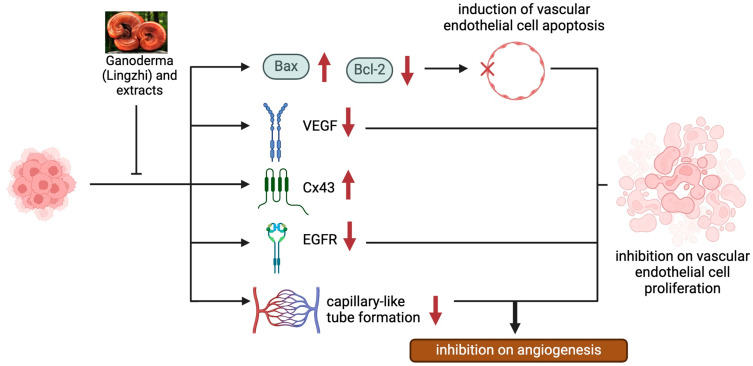
The anti-angiogenic effects of Lingzhi and its molecular mechanisms.

Angiogenesis supports tumor growth and progression by providing the oxygen and nutrients needed by rapidly growing cancer cells. Vascular endothelial growth factor (VEGF) represents the major angiogenesis inducer, facilitating the development of tumor vasculature. To this end, Lingzhi and Yunzhi have been reported as natural VEGF inhibitors (Figure 3 and Table 2), targeting tumor angiogenesis through multiple mechanisms. Lingzhi inhibits angiogenesis by downregulating VEGF expression and secretion through the suppression of the EGFR and PI3K/AKT/mTOR pathways [30]. It also reduces endothelial cell proliferation and capillary formation, thereby limiting tumor vascularization and oxygen/nutrient supply, which are crucial for tumor survival and metastasis [26,34,35,36].

Lingzhi polysaccharides (GLPS) have demonstrated the concentration-dependent inhibition of VEGF expression at both the mRNA and protein levels. In mouse melanoma cells (B16F10), GLPS reduced VEGF mRNA expression at concentrations as low as 0.2 µg/mL and protein levels at 0.8 µg/mL. A similar effect was also observed in human lung carcinoma cells (LA795) and hepatocarcinoma cells (HepG2), where GLPS significantly suppressed VEGF expression [11,37,38]. Additionally, *Ganoderma lucidum* polysaccharide peptide (GLPP) has also been reported to reduce VEGF secretion in human lung carcinoma cells (PG) at 200 µg/mL [21].

*Ganoderma lucidum* spore oil (GLSO) also demonstrated remarkable anti-VEGF activity. GLSO significantly decreased VEGF mRNA expression in HepG2 cells, human lung carcinoma cells (LTEP-a2), and breast carcinoma cells (MCF-7), with an approximately 50% reduction in VEGF expression in LTEP-a2 cells at 5–20 µg/mL [39,40,41]. In animal models, GLSO and spore powder effectively reduced tumor weight, volume, and VEGF levels in mice implanted with breast cancer cells (MDA-MB-231) and HCC cells (H22) at doses of 50–200 mg/kg [42,43,44,45]. These findings indicate the broad-spectrum anti-angiogenic properties of Lingzhi.

Similarly, polysaccharides derived from Yunzhi also demonstrated potent VEGF-inhibitory effects. Protein-bound polysaccharides (PBPs) were shown to regulate cytokine secretion to suppress VEGF production in murine breast cancer cells (4T1) at 50–200 µg/mL [47]. PSP significantly decreased tumor vascular density and VEGF mRNA expression in murine sarcoma models (S180) at 100–300 µg/mL [48]. In tumor xenograft models, *Coriolus versicolor* polysaccharide extract (CVE; 50–150 mg/kg) was shown to substantially reduced tumor weight and VEGF expression [49].

#### 3.1.3. Inhibition of Other Aberrant Oncogenic Pathways by Lingzhi/Yunzhi

Besides EGFR, Lingzhi was also reported to inhibit other oncogenic pathways, such as c-Met and Wnt/β-catenin, which are often involved in bypass resistance in EGFR-mutated NSCLC. Lingzhi-derived peptides (e.g., Lingzhi-8; LZ8) were found to suppress c-Met signaling to inhibit downstream pathways such as AKT and ERK [14], whereas Ganoderma extract was shown to block Wnt/β-catenin signaling by inhibiting LRP6 phosphorylation [50]. On the other hand, Lingzhi was also reported to reduce MMP-2 and MMP-9 expression, which are critical for tumor invasion and metastasis [51].

LZ8 and GLE, two bioactive components from *Ganoderma lucidum*, exhibited distinct antitumor mechanisms in different cancer types. LZ8 is an immunomodulatory protein isolated from the mycelial extract of Lingzhi [52]. It was shown to effectively inhibit cell migration and growth in both c-Met-overexpressing (HCC372) and c-Met-negative (HCC329) HCC at concentrations of 10–50 μg/mL. In immunocompromised SCID mice, LZ8 (10–20 mg/kg) significantly reduced primary HCC329 tumor growth and intrahepatic metastasis, with comparable potency to a c-Met-specific antagonist (JNJ-38877605). Mechanistically, LZ8 (20–100 μg/mL) effectively suppressed the phosphorylation of JNK, ERK, and AKT in c-Met-negative HCC329 cells. On the other hand, LZ8 was also found to reduce c-Met expression and phosphorylation, as well as the downstream p-ERK and p-AKT (but not p-JNK), in the c-Met-overexpressing HCC372 cells. Based on a receptor array screening of the major receptor tyrosine kinases, LZ8 demonstrated the most potent inhibitory effect on EGFR. In HepG2 cells, LZ8 suppressed HGF-induced c-Met signaling (p-c-Met, p-JNK, p-ERK, and p-paxillin) and cancer cell migration at concentrations of 25–100 μg/mL, outperforming JNJ-38877605 (a c-Met-specific antagonist). These findings highlight the anti-HCC potential of LZ8 via both c-Met-dependent and -independent pathways [14].

A commercially available whole-mushroom *Ganoderma lucidum* extract (GLE; manufactured by Pharmanex) has been reported to inhibit cancer proliferation and stemness in triple-negative breast cancer (TNBC) by targeting the STAT3 signaling pathway [53]. GLE was characterized as containing 5% polysaccharides, 6% triterpenes, and 1% cracked spores. At 50–200 μg/mL, GLE reduced the phosphorylation of STAT3 at Tyr705, a critical modification for STAT3 activation, in TNBC cell lines (MDA-MB-231 and SUM-149). This downregulation led to the decreased expression of a few key STAT3-regulated self-renewal transcription factors, including OCT4, NANOG, and SOX2. Additionally, GLE (50–250 μg/mL) also inhibited upstream components of the STAT3 pathway, such as JAK1 and JAK2. By disrupting STAT3 signaling, GLE (100–250 μg/mL) reduced the abundance of breast cancer stem cells (i.e., ALDH1-positive and CD44+/CD24− cell population) and inhibited mammosphere formation [53].

Lingzhi also suppressed oncogene-driven metastasis in EGFR-mutated NSCLC by targeting EMT and migration-related pathways. rLZ-8 inhibited EMT by downregulating a key transcription factor, Slug, but increasing E-cadherin expression, thereby suppressing cell mobility [54]. Furthermore, Lingzhi inhibited FAK-SRC-paxillin signaling, which disrupts cytoskeletal reorganization and focal adhesion formation, resulting in reduced tumor migration and invasion [26,55].

#### 3.1.4. Circumvention of Other EGFR TKI Resistance Mechanisms

Lingzhi was also reported to reactivate mutant p53, restoring its tumor-suppressive functions, and to reverse MDR by inhibiting P-gp, thereby increasing intracellular drug accumulation. These effects enhance the efficacy of chemotherapy and targeted therapies in resistant EGFR-mutant NSCLC [26,36,56] (summarized in Table 3).

Telomerase activation is associated with uncontrolled cell proliferation and represents a key cancer hallmark. Interestingly, the inhibition of telomerase, via the c-Myc-dependent downregulation of hTERT, has been recently shown to contribute to the antitumor activity of osimertinib [62]. The combination of a telomere-targeting inhibitor (6-Thio-dG) and osimertinib was shown to inhibit the growth of osimertinib-resistant NSCLC and to delay the emergence of acquired resistance to osimertinib. In this context, Lingzhi has been shown to inhibit telomerase activity by downregulating hTERT (the catalytic subunit of telomerase). This inhibition is mediated by suppressing the c-Myc transcription factor, which binds to the *hTERT* promoter [63]. In addition, some Lingzhi-derived compounds stabilize G-quadruplex structures in telomeric DNA, further inhibiting telomerase activity and reducing the ability of cancer cells to maintain their telomere length [26,64,65].

The Chinese herbal prescription Xiaoji decoction (XJD), which contains 15 g of Yunzhi, has been traditionally used for cancer therapy. Wu et al. reported that XJD effectively inhibited the growth of NSCLC cells through the ERK1/2-mediated reciprocal repression of HOTAIR and SP1 protein expression, subsequently leading to reduced EP4 gene expression. XJD also displayed synergistic antitumor effects with gefitinib in drug-resistant NSCLC cells, both in vitro and in vivo [61].

## 4. Involvement of Lingzhi/Yunzhi-Mediated Antitumor Immunity

### 4.1. Immunological Characteristics of EGFR-Mutated NSCLC

**Figure 4:**
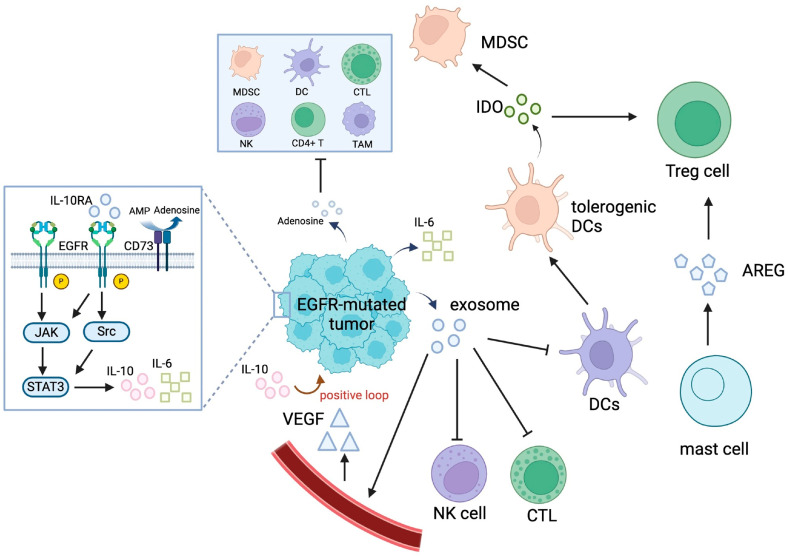
The immunosuppressive TME in EGFR-mutated NSCLC. Abbreviations: AMP, adenosine monophosphate; AREG, amphiregulin; CTL, cytotoxic T lymphocyte; DC, dendritic cell; GSK-3β, glycogen synthase kinase 3β; IDO, indoleamine 2,3-dioxygenase; IL-10, interleukin 10; IL-6, interleukin 6; MDSC, myeloid-derived suppressor cell; NK, natural killer; TAM, tumor-associated macrophage; TIL, tumor-infiltrating lymphocyte; TME, tumor microenvironment; Treg, regulatory T cell; VEGF, vascular endothelial growth factor.

The TME of EGFR-mutated NSCLC is characterized by a highly immunosuppressive and complex network of cells, cytokines, and signaling pathways that hinder effective antitumor immune responses (Figure 4) [5,66]. Compared to tumors harboring wild-type EGFR, EGFR-driven NSCLC tumors exhibit low tumor immunogenicity, including reduced TMB, fewer neoantigens, impaired T cell clonality, and lower PD-L1 expression.

#### 4.1.1. Low Tumor Immunogenicity

EGFR-mutated tumors typically have a lower TMB compared to EGFR wild-type tumors, resulting in fewer neoantigens and reduced immune recognition [67,68]. Moreover, EGFR-mutated tumors show impaired T cell expansion and weaker clonal diversity, reducing the ability of T cells to mount an effective antitumor response [69]. Furthermore, although EGFR mutations may upregulate PD-L1 through intrinsic signaling pathways (e.g., PI3K/AKT/mTOR, JAK/STAT), PD-L1 expression in EGFR-mutated tumors is generally lower than in EGFR wild-type tumors [70].

#### 4.1.2. EGFR-Mutated Tumors Are Infiltrated with Immunosuppressive Immune Cells

EGFR activation promotes the infiltration and activation of immunosuppressive regulatory T cells (Tregs), which suppress antitumor immunity [71,72]. Despite the presence of dendritic cells (DCs) in EGFR-mutated tumors, their maturation is suppressed by the IL-6/STAT3 signaling pathway, leading to impaired antigen presentation [6,73]. Moreover, tumor-associated macrophages (TAMs) in EGFR-mutated NSCLC exhibit an immunosuppressive M2 phenotype, secreting factors like IL-10 and VEGF, which promote tumor progression and immune evasion [6,72]. In addition, EGFR-mutated tumors often lack the sufficient infiltration of cytotoxic CD8+ T cells, and the T cells present are frequently inactivated or dysfunctional [74,75]. Furthermore, natural killer (NK) cells are less abundant and phenotypically anergic (i.e., lacking the ability to react to antigens) in EGFR-mutated tumors, partly due to high levels of TGF-β in the TME [76,77].

#### 4.1.3. EGFR-Mutated NSCLC Secretes Immunosuppressive Cytokines

IL-6 and IL-10 are known to be upregulated in EGFR-mutated tumors with acquired resistance to TKIs [73,76]. These immunosuppressive cytokines impair antitumor immunity by promoting Treg and myeloid-derived suppressor cell (MDSC) recruitment and inhibiting T and NK cells. EGFR-mutated tumors with activated EGFR signaling are known to secrete VEGF, thereby promoting angiogenesis and creating an immunosuppressive TME [6,78]. Moreover, EGFR activation has been reported to synergize with TGF-β signaling to promote EMT and suppress T cell infiltration [76,77]. Furthermore, the overexpression of CD73 in EGFR-mutated tumors is known to increase adenosine levels, thereby suppressing CD8+ T cell and NK cell activity [79,80]. Recently, exosomes secreted from EGFR-mutated tumors were shown to contain oncogenic EGFR and various immunosuppressive factors, which induce the apoptosis of CD8+ T cells, impair DC function, and promote Treg development [6,81].

#### 4.1.4. EGFR-TKI Treatment Reinforces the Immunosuppressive TME in EGFR-Mutated Tumors

Recent research has revealed a dynamic shift toward a more immunosuppressive TME in EGFR-mutated tumors upon EGFR-TKI therapy [73]. When treatment-naïve EGFR-mutated NSCLC was treated with TKIs, the initial treatment response was accompanied by an increase in cytotoxic CD8+ T cell infiltration but a reduction in immunosuppressive Tregs and M2-like TAMs [6,73,74]. EGFR-TKIs also upregulated MHC class I molecules on tumor cells, improving antigen presentation to T cells and enhancing immune recognition [74,76]. Initial treatment with EGFR-TKIs also reduced levels of immunosuppressive cytokines such as IL-6 and increased levels of interferon-gamma (IFN-γ), which supported immune activation [6,73]. However, this immune-permissive TME was only transient and gradually shifted to a more immunosuppressive state as treatment continued.

Intriguingly, upon extended EGFR-TKI treatment, EGFR-mutated tumors developed an immunosuppressive TME. There was an accumulation of MDSCs in the TME after prolonged EGFR-TKI therapy, which suppressed T cell activity [73]. Continuous EGFR-TKI treatment also increased the levels of IL-10 and CCL2, both of which contribute to an immunosuppressive environment [73]. While intratumoral CD8+ T cell infiltration initially increased, prolonged EGFR-TKI treatment led to decreased T cell density and activity, particularly in tumors with low PD-L1 expression [74]. Moreover, sustained EGFR-TKI therapy may also result in T cell exhaustion, reducing their ability to sustain effective antitumor immune responses [73].

### 4.2. Immunomodulatory Effects of Lingzhi/Yunzhi and Their Potential Role in Treating EGFR-Mutated NSCLC

**Figure 5:**
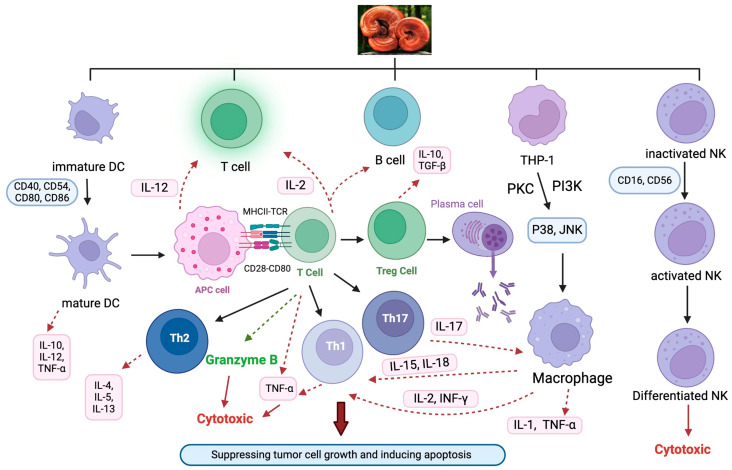
The immunomodulatory effects of *Ganoderma* on various immune cells. Abbreviations: APCs, antigen-presenting cells; DCs, dendritic cells; IL, interleukin; IFN-γ, interferon-γ; NK, natural killer cells; PKC, protein kinase C; Th, T helper cells; TNF-α, tumor necrosis factor-α; Treg, regulatory T cells.

The stimulatory effect of Lingzhi/Yunzhi on host immune systems plays a vital role in their antitumor effects (Figure 5). As there are overwhelmingly more investigations into the antitumor effect of Lingzhi than Yunzhi in the literature, most examples described below are devoted to Lingzhi. The relevance of EGFR-mutated tumors is discussed.

#### 4.2.1. Ganoderma Boosts Antitumor Immunity

##### Enhancing Antitumor Activity of Macrophages

Macrophages are the most abundant immune cells bearing multifaceted roles in regulating tumor growth in the TME. They are characterized by cellular plasticity, which can switch from an anti-inflammatory/tumor-permissive (M2) to a pro-inflammatory/antitumor (M1) phenotype, depending on the changes in the TME [82]. Therefore, macrophages represent an attractive target for cancer immunotherapy. It is feasible to inhibit tumor proliferation by reprogramming the macrophages within the TME towards the antitumor M1 phenotype or preventing the adoption of the pro-tumor M2 phenotype [83].

Macrophages are known to eliminate tumor cells by phagocytosis, after recognizing the “eat-me” plasma membrane proteins on tumor cells [84]. To this end, Lingzhi has been reported to enhance the recognition of pathogens by macrophages through the increased expression of various surface receptors. Wei et al. reported that *G. lucidum* polysaccharides (EORP, 50–200 μg/mL) upregulated the expressions of two surface receptors on macrophages (toll-like receptor 4 (TLR4) and CD14), thus improving their ability to recognize and respond to LPS to kill tumor cells [85]. In a mouse study, *G. lucidum* extract and polysaccharide D6 were shown to markedly improve the phagocytic ability of peritoneal macrophages [86]. A specific bioactive proteoglycan fraction from the fruiting body of *G. lucidum* (GLIS) was also reported to enhance macrophage phagocytosis in tumor-bearing mice [87]. GLIS (200 mg/kg) was found to increase macrophage-mediated phagocytosis and antitumor activity in a sarcoma S180 mouse model, resulting in a 60% reduction in tumor growth [88].

Moreover, Lingzhi was also known to stimulate the secretion of pro-inflammatory cytokines from macrophages. Various *Ganoderma* extracts and *G. lucidum* spore polysaccharides (50–200 μg/mL) have been shown to promote IL-1 production in mice [89,90]. GLIS was also reported to stimulate the production of TNF-α, IL-1, and nitric oxide (NO) in the TME of tumor-bearing mice [87]. Recently, GLIS (50–200 μg/mL) was also reported to activate bone marrow-derived macrophages in tumor-bearing mice, which secreted the pro-inflammatory cytokines IL-1β, TNF-α, and NO to trigger higher antitumor activities [88]. PSG-1, a polysaccharide derived from *G. atrum*, was found to promote TNF-α transcription and macrophage activation via the NF-κB, PI3K/Akt, and MAPK pathways in a concentration-dependent manner (50–200 μg/mL) [91].

On the other hand, polysaccharide from *Ganoderma lucidum* (PS-G) was known to activate key intracellular signaling pathways (PKC, p38 MAPK, and Src-family kinases such as Lyn) in macrophages, which enhanced phagocytosis and the migration of neutrophils [92,93]. PS-G also inhibited apoptosis via Akt-regulated signaling pathways. Moreover, *Ganoderma atrum* polysaccharide (PSG-1) (50–200 μg/mL) was reported to activate macrophages through TLR4-dependent pathways, leading to NF-κB activation and increased cytokine production [91]. Furthermore, triterpene-rich extracts from *G. lucidum* (AF) (50–100 μg/mL) enhanced TNF-α production by modulating p38 and JNK MAPK pathways [94].

Lingzhi was also reported to promote the M1 polarization of macrophages to enhance antitumor immunity. Sun et al. observed that *G. lucidum* polysaccharides (GL-PS) promoted M1 polarization in the presence of LPS, which was accompanied by an increase in TNF-α, IL-6, and IL-12 production but a decrease in IL-10 secretion [95]. Similarly, β-glucans from *G. lucidum* (50–200 μg/mL) were shown to upregulate the M1 phenotype markers (e.g., IL-12, IFN-γ) in tumor-associated macrophages but reduce the M2 phenotype markers (e.g., TGF-β, IL-10) in the TME [96].

Last but not least, upon the exposure of macrophages to tert-butyl hydroperoxide and alloxan-induced oxidative damage, *G. lucidum* polysaccharide peptide (GLPP, 50–200 μg/mL) prevents macrophage necrosis by reducing free radical levels and preserving mitochondrial membrane potential [17,19].

##### Enhancing the Antitumor Activity of NK Cells

NK cells are specialized immune effector cells that play critical roles in tumor immunosurveillance by producing cytokines and secreting lytic granules [97]. Lingzhi has been shown to promote NK cell activation and differentiation of other immune cells under immunosuppressed conditions. The oral administration of crude Lingzhi extract (3–6 mg/kg) was reported to significantly increase NK cell activity in mouse models of WEHI-3 leukemia cells [98]. The extract promoted the activation and proliferation of NK and T cells and significantly reduced the abundance of leukemia cells in the spleen. Lingzhi polysaccharides were also reported to activate NK cells through TLR4 signaling [99]. Tsai et al. demonstrated that acid-hydrolyzed polysaccharide fragments of Lingzhi (GLPS-SF1 and GLPS-SF2) stimulated NK cell activation and proliferation. GLPS-SF1 (50–200 μg/mL) was found to induce the production of antitumor cytokines (IL-12, TNF-α, and IFN-γ) via TLR4 signaling. On the other hand, GLPS-SF2 (an oligosaccharide fraction; 50–200 μg/mL) was shown to promote NK and T cell activation and proliferation. The unique sugar structures of these polysaccharides were shown to mediate immune-stimulating effects [99].

Lingzhi also enhanced NK cell cytotoxicity by increasing their functional activity. Won et al. demonstrated that alcohol-insoluble components (GL-AI) of Lingzhi significantly enhanced the cytotoxic activity of spleen NK cells in C3H/HeN mice when administered through oral (100–500 mg/kg), intraperitoneal (i.p., 4–200 mg/kg), or intravenous (i.v., 1–50 mg/kg) routes. Similarly, water-soluble extracts of *Ganoderma tsugae* mycelium (GT) and its alcohol-insoluble fraction (GTI, 1–50 mg/kg) increased NK cell activity in a dose-dependent manner, along with elevated blood interferon (IFN) levels. Neutralization studies confirmed that both IFN-α/β and IFN-γ were critical to this enhanced NK activity [9,100]. Ning et al. reported that the intraperitoneal injection of Lingzhi polysaccharides (1 mg/day) for 7 days significantly increased NK cell activity, lymphocyte proliferation, and serum levels of TNF-α and IL-2 in tumor-bearing mice. These cytokines are essential for activating NK cells and promoting antitumor immunity [101].

Under immunosuppressive conditions, Lingzhi was shown to restore the antitumor functions of NK cells. In mice treated with the chemotherapeutic drug cyclophosphamide, low doses of Lingzhi polysaccharides (2.5 mg/kg; daily for 7 days) were found to enhance NK cell cytotoxicity, lymphokine-activated killer (LAK) cell activity, and macrophage phagocytosis [10]. Lingzhi polysaccharides also accelerated the recovery of bone marrow cells and T and B lymphocyte proliferation without side effects, suggesting its potential as an adjunct therapy to mitigate chemotherapy-induced immunosuppression [10].

##### Modulating T Cells to Enhance Antitumor Response

Lingzhi is known to promote antitumor immunity by modulating T cells through various mechanisms, including enhancing T cell activation, promoting the differentiation of T cell subsets, and regulating cytokine production. Studies in glioma-bearing rats demonstrated that Lingzhi polysaccharides (Gl-PS) significantly elevated these cytokines, which are critical for T cell activation and immune responses [102]. A few other studies also revealed that Gl-PS boosted the mRNA and protein expression of IL-2, IFN-γ, and TNF-α in splenic T cells while augmenting their antitumor cytotoxicity [103,104,105]. Moreover, GLE (oral administration at 200–400 mg/kg) promoted T cell receptor signaling pathways and increased CD3^+^, CD4^+^, and CD8^+^ T cell subsets in tumor-bearing mice [106].

Intriguingly, Lingzhi was also found to restore the balance between CD4^+^ helper T cells and CD8^+^ cytotoxic T cells. In NSCLC patients undergoing chemotherapy, *Ganoderma lucidum* spores (oral administration at 1.5 g/day) increased CD4^+^ T cell levels and the CD4^+^/CD8^+^ ratio, suggesting improved cellular immunity [107]. Similarly, in HCC patients after surgery, Lingzhi spores (1.5 g/day) were also found to elevate CD4^+^ T cells and restore immune homeostasis [108]. The polysaccharide purified from *Ganoderma lucidum* (PS-G, 10 µg/mL) was reported to promote antitumor Th1 responses by increasing IFN-γ production through dendritic cell activation and IL-12 secretion [109].

Lingzhi modulates cytokine production by promoting the release of pro-inflammatory cytokines while reducing immunosuppressive ones. Lingzhi polysaccharides (50–200 μg/mL) stimulated T lymphocytes to release IL-2 and IFN-γ, thereby enhancing T cell activation and cytotoxicity against tumor cells [110]. In tumor-bearing mice, Lingzhi extract (GLE; 200–400 mg/kg) was known to upregulate cytokines such as IFN-γ, IL-2, and TNF-α, thus producing T cell-mediated antitumor immunity [106]. Furthermore, *Ganoderma lucidum* polysaccharides (Gl-PS; at concentrations of 0.8, 3.2, and 12.8 µg/mL) have been reported to reduce the levels of two immunosuppressive cytokines (IL-10 and TGF-β1), which are often secreted by tumors to evade immune surveillance [11,12].

It is particularly noteworthy that Lingzhi could restore T cell function in immunosuppressed cancer patients. In chemotherapy-treated patients, Lingzhi extract (16 capsules/day) prevented reductions in CD3^+^, CD4^+^, and CD8^+^ T cells, which are crucial for cellular immunity [111]. Similarly, in HCC patients after surgery, Lingzhi spores (1.5 g/day) have been shown to improve T cell numbers and restore immune balance, presumably preventing tumor recurrence [108]. The metabolic or functional exhaustion of T cells represents an important drug resistance mechanism to immune checkpoint blockade therapy [112]. Recently, a water extract from sporoderm-breaking spores of *Ganoderma lucidum* (ESG) (oral administration, 400 mg/kg) was shown to downregulate the immune checkpoint molecules PD-1 and CTLA-4, thus restoring T cell functionality and enhancing tumor immune surveillance in tumor-bearing mice [113].

Collectively, Lingzhi modulates T cells by enhancing their activation, regulating cytokine production, balancing T cell subsets, restoring immune function in immunosuppressed conditions, and suppressing immune checkpoint molecules. Thus, Lingzhi represents a promising adjunct therapy for enhancing antitumor immunity during cancer treatment.

### 4.3. Potential Role of Lingzhi in EGFR-Mutated NSCLC: A Tumor Immunology Perspective

As discussed above, EGFR-mutated NSCLC is characterized by an immunosuppressive TME, leading to tumor evasion from immune surveillance. Accumulating evidence suggests that Lingzhi could be used as an immunomodulatory adjuvant in EGFR-mutated NSCLC to enhance therapeutic outcomes. By enhancing dendritic cell function, activating T cells and NK cells, suppressing immunosuppressive cells, and regulating cytokines, the integration of Lingzhi into existing therapies could enhance immune responses, reduce resistance, and improve clinical outcomes for patients with EGFR-mutated NSCLC [11,109,113]. In addition, Lingzhi was also found to promote macrophage polarization toward the M1 phenotype and facilitate antitumor immunity, but it inhibited pro-tumor M2 macrophage polarization [95].

In EGFR-mutated NSCLC, immunosuppressive cytokines such as IL-6, VEGF, and TGF-β are elevated, which contribute to immune evasion and tumor progression. Lingzhi was able to restore immune balance by increasing pro-inflammatory cytokines like IFN-γ, IL-12, and TNF-α, which activate cytotoxic T cells and NK cells while decreasing immunosuppressive cytokines like IL-10, TGF-β, and VEGF [11,12,106]. By modulating cytokine levels, Lingzhi creates a more favorable immune environment for antitumor activity.

NK cells play a critical role in tumor suppression but are often phenotypically inactive in EGFR-mutated NSCLC due to high levels of TGF-β in the TME [114]. Lingzhi has been shown to increase NK cell numbers, boost their cytotoxic functions by increasing perforin and granzyme production, and restore NK cell effectiveness, which is critical for overcoming immune evasion [9,10,100].

The adenosine signaling pathway, driven by CD73 overexpression, is a key immunosuppressive mechanism against T cells and NK cells in EGFR-mutated NSCLC [78]. Lingzhi has been shown to downregulate CD73 expression and reduce adenosine levels, thereby restoring antitumor immunity [12,115].

EGFR-mutated NSCLC patients generally respond poorly to immune checkpoint inhibitors (ICIs) due to the immunosuppressive TME [116]. Lingzhi’s immunomodulatory properties, including enhanced T cell infiltration, reduced Tregs and MDSCs, and cytokine modulation, may improve the efficacy of ICIs. These effects position Lingzhi as a potential adjuvant therapy to overcome resistance and enhance immune responses in EGFR-mutated NSCLC [106,113]. While EGFR TKIs are standard treatment for EGFR-mutated NSCLC, their prolonged use can lead to an immunosuppressive TME [117]. Lingzhi may complement EGFR TKIs by enhancing immune surveillance, preventing the accumulation of suppressive immune cells like MDSCs, and maintaining an active immune response [11,113]. Therefore, Lingzhi may be used as an adjuvant to facilitate the efficacious antitumor combination of EGFR TKIs and immune checkpoint blockade therapy. Recently, Lingzhi was also reported to counteract other immunosuppressive mechanisms in EGFR-mutated NSCLC, such as exosome-mediated immune modulation and impaired antigen presentation. By enhancing MHC-I expression and reducing T cell apoptosis, Lingzhi could improve tumor immune recognition [11,103]. Furthermore, the capacity of Lingzhi to modulate immune cell markers and cytokine profiles may reduce the risk of hyperprogressive disease, which is characterized by a paradoxical rapid tumor growth after initial treatment with PD-1/PD-L1 immunotherapy [118].

## 5. Pharmacokinetics Consideration

### 5.1. Potential Lingzhi–EGFR TKI Interaction via Drug Transporters

EGFR-TKIs are substrates of key drug transporters including P-glycoprotein (P-gp), breast cancer resistance protein (BCRP), and multidrug resistance proteins (MRPs) [119]. These transporters restrict drug accumulation in various organs/tissues, such as the brain. The effects of Lingzhi on various drug transporters are summarized in Table 4. Cao et al. conducted a detailed investigation on the effects of *Ganoderma lucidum* extract (GLE), *Ganoderma lucidum* polysaccharides (GLP), or *Ganoderma lucidum* triterpenoids (GLT) (in particular, ganoderic acids; GAAs) on the transport activity of P-gp, MRP, and BCRP [119]. The results from the cellular uptake experiment showed that GLE (100 μg/mL) could significantly increase the cellular accumulation of P-gp or MRP substrate (Rhodamine 123 (Rho) and Calcein (Cal), respectively) by inhibiting the efflux transporter functions. In contrast, GLE was found to significantly decrease the cellular accumulation of BCRP substrate Hoechst 33342 (Hoe) by inducing BCRP transport activity. On the other hand, GLP did not show any significant effects on P-gp or MRP but induced the drug efflux activity of BCRP. GAA did not affect the activity of the three efflux transporters at the experimental concentrations [119].

Using a bidirectional Caco-2 permeability assay, the transport of specific transporter probe substrate (Rho or Cal) was measured in the apical to the basolateral direction (AP→BL) in the presence or absence of GLE [119]. The apparent permeability (Papp A-B) values could be calculated to assess the inhibition of specific drug transporters by GLE. GLE was shown to significantly increase (AP→BL) transport as well as Papp A-B values for the P-gp substrate (Rho) and MRP substrate (Cal), suggesting its inhibition of P-gp/MRP-mediated drug efflux [119]. Among all EGFR-TKIs, osimertinib displays the lowest efflux ratio, suggesting that it is less affected by drug transporters [123]. While gefitinib, erlotinib, afatinib, and osimertinib are all considered to be P-gp substrates in vitro, patient data indicated that clinically relevant drug–drug interactions are only prominent for the second-generation EGFR TKI afatinib [124].

The overexpression of ABC transporters in cancer cells is known to mediate MDR to numerous chemotherapeutic drugs [125]. Recently, a triterpenoid (Ganoderiol F) isolated from Lingzhi was reported to inhibit P-gp and reverse P-gp-mediated multidrug resistance by increasing the intracellular accumulation of a P-gp substrate anticancer drug doxorubicin [126]. While EGFR-TKIs are known substrates for major ABC drug transporters, the circumvention of ABC transporter-mediated drug resistance to EGFR-TKIs by Lingzhi/Yunzhi in experimental cancer models has not been reported.

### 5.2. Potential Lingzhi/Yunzhi-EGFR TKI Interaction via Metabolic Enzymes

The four clinically approved EGFR-TKIs (gefitinib, erlotinib, afatinib, and osimertinib) are metabolized by cytochrome P450 (CYP) enzymes, particularly CYP3A4, thus making them susceptible to drug–drug interactions involving CYP modulators [124]. The potential modulatory effect of Lingzhi/Yunzhi on various CYP enzymes is summarized in Table 5. While Lingzhi/Yunzhi polysaccharides (such as GLP and PSP) display limited CYP inhibition, the triterpenoids component (e.g., ganoderic acid A (GAA)) has been reported to exhibit a more significant CYP inhibitory effect [127]. In general, the likelihood of herb–drug interactions appears low for most Lingzhi/Yunzhi preparations but may be clinically relevant for drugs metabolized by CYP3A4 or when using concentrated triterpenoids like GAA.

In human liver microsomes (HLMs), GLE exhibited weak inhibitory effects on CYP2C19, 2D6, 3A4, and 2C9 with IC_50_ values of 131.2, 164.4, 150.5, and 142.2 μg/mL, respectively. The inhibitory effect of GLT was slightly stronger than that of GLE with IC_50_ values of 102.5, 116.1, 136.4, and 82.2 μg/mL, respectively [119]. On the other hand, GLP did not significantly affect the six CYP450 enzymes. In rat liver microsomes (RLM), GLT showed a weak inhibitory effect on CYP2C9 with an IC_50_ value of 163.1 μg/mL but did not affect the activity of other enzymes. In a Sprague Dawley rat study, the inhibitory effect of GLP on CYP1A2, CYP3A4, and CYP2E1 was evaluated by analyzing the metabolism of the corresponding enzyme substrate drugs (phenacetin, nifedipine, and chlorzoxazone, respectively) [128]. GLP was shown to inhibit the three CYP enzymes in a concentration-dependent manner, suggesting possible herb–drug interactions between GLP and other concomitantly administered CYP substrate drugs.

GAA inhibited the activity of CYP3A4, 2D6, and 2E1 but did not affect other isoforms. The inhibition of CYP3A4, 2D6, and 2E1 was concentration-dependent with IC_50_ values of 15.05, 21.83, and 28.35 μM, respectively. Additionally, GAA was not only a non-competitive inhibitor of CYP3A4 but also a competitive inhibitor of CYP2D6 and 2E1, with Ki values of 7.16, 10.07, and 13.45 μM. Meanwhile, the inhibition of CYP3A4 was time-dependent, with a K_I_ /K_inact_ value of 7.91/0.048 μM/min. This in vitro study indicated that GAA has the potential to result in drug–drug interactions with other drugs metabolized by CYP3A4, 2D6, and 2E1. Further clinical studies are needed for the identification of this interaction [130].

Polysaccharide peptide (PSP), isolated from the COV-1 strain of *Coriolus versicolor* (Yunzhi), can competitively inhibit tolbutamide 4-hydroxylation in both pooled human liver microsomes and specific human CYP2C9 in vitro. However, considering the comparatively high Ki values observed for PSP interaction with human CYP2C9, it is unlikely to significantly contribute to herb–drug interactions [129,132]. “I’m-Yunity” is a herbal supplement produced from a specific strain of Yunzhi. PSP is believed to be the active ingredient in I’m-Yunity. To assess the impact of I’m-Yunity on CYP3A4, Nicandro et al. conducted an open-label study involving 12 healthy adult volunteers, providing participants with a 14-day supply of I’m-Yunity and instructing them to take 1200 mg thrice daily with meals. The results indicated that the short-term use of I’m-Yunity for 14 days did not lead to any significant inhibition or induction of CYP3A4. The concurrent administration of I’m-Yunity with medications primarily metabolized by CYP3A4 is unlikely to result in significant herb–drug interactions [131].

Collectively, Lingzhi exhibits pharmacokinetic interactions with EGFR-TKIs by modulating efflux transporters and CYP enzymes, potentially enhancing drug efficacy but requiring careful monitoring to avoid adverse effects. On the other hand, Yunzhi offers a lower risk profile for pharmacokinetic interactions, making it an attractive complementary therapy. Further clinical studies are necessary to validate these findings and optimize the use of Lingzhi and Yunzhi in combination with EGFR-TKIs for improved cancer treatment outcomes.

## 6. Conclusions and Future Perspectives

This review highlights the significant potential of Lingzhi and Yunzhi as complementary therapeutic agents in managing EGFR-mutated NSCLC. The bioactive components in Lingzhi and Yunzhi, such as polysaccharides and triterpenoids, exhibit multifaceted antitumor properties. These include the inhibition of oncogenic signaling pathways (e.g., PI3K/AKT/mTOR, ERK, Wnt/β-catenin), suppression of EMT, reactivation of tumor suppressors (p53), and reversal of multidrug resistance [14,15,56]. Moreover, the immunomodulating effects of Lingzhi/Yunzhi, including enhanced activity of T cells and NK cells, reduced Tregs and MDSCs, and suppression of immunosuppressive cytokines (e.g., IL-6, VEGF, and TGF-β) further support their potential to complement immune checkpoint blockade therapy in EGFR-mutated NSCLC [9,10,11,12].

Despite these promising findings, challenges and knowledge gaps remain. Current preclinical and limited clinical studies highlight the efficacy of Lingzhi and Yunzhi, but large-scale, randomized clinical trials are needed to confirm their safety, efficacy, and optimal integration with EGFR-TKIs and ICIs in NSCLC treatment. Importantly, potential pharmacokinetic interactions between these medicinal mushrooms and conventional therapies require further exploration to ensure safe and effective combination regimens [15,131,133]. Lingzhi, with its triterpenoids, inhibits key efflux transporters like P-gp and MRPs, which could increase the tissue retention of concurrently administered antitumor drugs [13,119]. Moreover, Lingzhi also exhibits mild inhibitory effects on CYP enzymes (e.g., CYP3A4, CYP2D6), which may alter drug metabolism and clearance of co-administered EGFR-TKIs [130]. In contrast, the bioactive ingredient of Yunzhi (polysaccharopeptides, PSP) exhibits negligible effects on drug transporters and CYP enzymes, offering a safer pharmacokinetic profile [129,131].

Future research should prioritize well-designed clinical trials to validate the efficacy and safety of Lingzhi and Yunzhi as adjuvants in NSCLC treatment. An excellent systematic review of clinical studies investigating medicinal mushroom supplements for cancer therapy has been published recently [134]. There were 12 different mushroom preparations reported in the 39 trials included in that systematic review. In two hepatocellular carcinoma studies [135,136] and one breast cancer study [137] investigating Huaier granules (*Trametes robiniophila* Murr), patient survival benefit was reported. In combination with chemotherapy, polysaccharide K (PSK) was also shown to produce survival benefit in four gastric cancer studies [138,139,140,141]. Some of these trials only investigate the antitumor effect of the mushrooms alone, not their combinations with other chemotherapeutic drugs. Moreover, the sample size was small in some trials, and they did not use a randomized controlled trial design. Therefore, the evidence so far is inconclusive to recommend the routine use of medicinal mushrooms for treating cancer patients. These trials should evaluate key clinical endpoints, such as progression-free survival (PFS), overall survival (OS), and quality of life (QoL), in patients receiving combinations of EGFR-TKIs and these medicinal mushrooms. Indeed, a multicenter, randomized, double-blind, placebo-controlled clinical trial (China Clinical Trial Registry ChiCTR2300072786) is currently recruiting patients with an aim to investigate the potential synergistic antitumor effect of de-walled *Ganoderma Lucidum* spore powder on targeted therapy in advanced NSCLC patients [142]. Further mechanistic studies are needed to elucidate the molecular interactions between Lingzhi/Yunzhi and EGFR pathways, resistance mechanisms (e.g., *MET*/*HER2* amplification), the TME, and systemic immunity.

Moreover, personalized approaches should be explored, focusing on genetic polymorphisms in metabolic enzymes and drug transporters to predict individual patient responses to Lingzhi/Yunzhi-based combination therapy with osimertinib. Predictive biomarkers of therapeutic response should also be identified to optimize treatment for specific patient subgroups. To this end, a recent clinical study demonstrated that a few single nucleotide polymorphisms (SNPs) in CYP enzymes (CYP2A6*4 and CYP2C9*3) were associated with the increased incidence of adverse drug reactions and also reduced median PFS in Thai patients with NSCLC on osimertinib therapy [143]. Intriguingly, another recent clinical study revealed that SNPs of two efflux transporters (ABCB1 3435C>T and ABCG2 34G>A) were significantly correlated with the development of brain metastases in patients with NSCLC on osimertinib therapy, probably due to diminished intracerebral drug levels [144]. On the other hand, the result from a retrospective observational multicenter study showed that the ABCG2 421C>A genotype was associated with a higher incidence of diarrhea in patients with NSCLC on osimertinib therapy [145]. As Lingzhi and Yunzhi may modulate the pharmacokinetic properties of osimertinib via these CYP enzymes and/or ABC transporters, pharmacogenetic testing may facilitate the selection of a patient subgroup to benefit from the herb–drug combination. In addition, combination regimens involving Lingzhi/Yunzhi, EGFR-TKIs, ICIs, or other targeted therapies should be evaluated. Such synergistic approaches may overcome drug resistance, enhance antitumor immunity, reduce toxicities, and improve outcomes in EGFR-mutated NSCLC.

Standardization and quality control of Lingzhi and Yunzhi products are critical to ensure consistent pharmacological effects in clinical applications. Future research should focus on identifying active components, optimizing extraction methods, and maintaining rigorous quality standards [146,147]. Given their broad-spectrum antitumor and immunomodulatory effects, Lingzhi/Yunzhi may be applicable to other EGFR-driven malignancies, such as head and neck cancers and inflammatory breast cancer [14,15,23].

In conclusion, Lingzhi and Yunzhi represent a promising advancement in integrative cancer therapy, particularly for addressing treatment resistance and immunosuppression in EGFR-mutated NSCLC. Their successful clinical integration will require concerted efforts in clinical research, standardization, and mechanistic studies to ensure efficacy, safety, and quality as adjuvant therapies.

## Figures and Tables

**Table 1:** Modulation of EGFR signaling by Lingzhi.

Species	Herbal Component Contributing to the Biological Effect	Study Systems (Cell, Microsome, Animal)	Major Effects	Reference
*Ganoderma lucidum*	WSG	Human lung adenocarcinoma A549 cell line and murine Lewis lung carcinoma (LLC1)	(1) WSG significantly suppressed the viability and motility of lung cancer cells. (2) WSG markedly inhibited lung tumor progression, decreased the size of metastatic nodules within the lungs, and extended the survival of LLC1-bearing mice.	[7]
*Ganoderma lucidum*	WSG	Human tongue cancer SAS and HSC3 cells	(1) The IC_50_ values for SAS and HSC3 cells following 48 h treatment with WSG were 107 μg/mL and 232 μg/mL, respectively. (2) WSG did not significantly reduce the EGFR level but did significantly reduced EGFR phosphorylation by about 50–60%. (3) WSG + cisplatin enhanced cytotoxicity in tongue cancer cells.	[27]
*Ganoderma lucidum*	rLZ-8	Human lung cancer cell lines; Lewis tumor-bearing C57BL/6 mice	(1) rLZ-8 significantly suppressed EGFR protein level. (2) rLZ-8 significantly decreased (i) tumor weight and volume. (ii) EGFR expression in tumor lesions.	[15]
*Ganoderma lucidum*	rLZ-8	Hep3B, A549, MDA-MB-468 and B16F10 cancer cells; Orthotopic HCC NOG mouse models. Patient-derived tumor xenograft (PDX) models of HCC (LI6280, LI1097, LI0050, LI0334, LI6611)	(1) Cancer cell growth (Hep3B, A549, MDA-MB-468, and B16F10 cancer cell lines) was inhibited significantly in vitro. (2) rLZ-8 exhibited a dose-dependent inhibition of tumor xenograft growth. (3) The tumor inhibitory efficacy of rLZ-8 was directly correlated with EGFR expression levels.	[28]
*Ganoderma lucidum*	EGDT 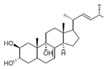	Well and poorly differentiated NPC cell lines CNE1 and CNE2 BALB/C nu/nu female mice	(1) EGDT reduced the protein and mRNA levels of EGFR as well as its downstream RAF/MEK/ERK and PI3K/AKT signaling pathways in a time- and dose-dependent manner. (2) EGDT demonstrated significant antitumor activity in NPC xenograft models in vivo.	[29]
*Ganoderma tsugae*	GTME	The human epidermoid carcinoma A431 cell line BALB/C nu/nu nude mice	GTME suppressed EGFR expression, leading to reduced VEGF secretion in epidermoid carcinoma A431 cells through the inhibition of VEGF expression.	[30]
*Ganoderma lucidum*		Adult male Sprague Dawley rats	GL attenuated the EGFR expression in cisplatin-exposed renal tissues.	[31]
*Ganoderma lucidum*	GPS-49 and GSPS-49	Lung cancer A549 and LLC1 cells	At 49 days, GPS and GSPS demonstrate significant anticancer effects without impacting normal fibroblasts. GPS-49 and GSPS-49 inhibit lung cancer by targeting and suppressing the signaling networks mediated by epidermal growth factor receptor (EGFR) and transforming growth factor beta receptor (TGFβR).	[32]

Abbreviations: WSG: a water-soluble glucose-enriched polysaccharide from *Ganoderma lucidum*; rLZ-8: recombinant Ling Zhi-8; EGDT: Ergosta-7,22-diene-2*β*,3*α*,9*α*-triol; NPC: nasopharyngeal carcinoma; GTME: *G. tsugae* methanol extract; GPS-49: *Ganoderma lucidum* polysaccharides isolated on day 49; GSPS-49: *Ganoderma lucidum* sulfated polysaccharides isolated on day 49.

**Table 2:** Modulation of VEGF-dependent angiogenesis program by Lingzhi/Yunzhi.

Species	Herbal Preparation	Study Systems (Cell, Microsome, Animal)	Major Effects	Reference
*Ganoderma lucidum*	Extract	Human prostate cancer cell line PC3	GLE significantly inhibited (i) capillary morphogenesis; (ii) VEGF secretion in a dose-dependent manner.	[20]
*Ganoderma lucidum*	Polysaccharide peptide (GLPP)	Human lung carcinoma cells PG	GLPP significantly inhibited VEGF secretion at 200 μg/mL.	[21]
*Ganoderma lucidum*	Extract	Human ovarian cancer cells HO8910 (HOCCs) Human primary ovarian cells (HPOCs)	GLE significantly decreased VEGF: mRNA expression and protein level in HOCC in dose-dependent manner; mRNA expression in HPOC.	[22]
*Ganoderma lucidum*	Polysaccharide	Mouse melanoma cells B16F10	GLPS significantly decreased VEGF: mRNA expression from 0.2 μg/mL; Protein level from 0.8 μg/mL.	[11]
*Ganoderma lucidum*	Polysaccharide	Mouse melanoma cells B16F10	GLPS significantly decreased VEGF mRNA expression and protein level from 0.8 μg/mL.	[37]
*Ganoderma lucidum*	Polysaccharide	Human hepatocarcinoma cells HepG2	GLPS significantly decreased VEGF protein level after 24 h of incubation.	[38]
*Ganoderma lucidum*	Spore oil	Human hepatocarcinoma cells HepG2	GLSO significantly decreased VEGF mRNA expression.	[39]
*Ganoderma lucidum*	Spore oil	Human breast carcinoma cells MCF-7	GLSO slightly decreased VEGF mRNA expression.	[40]
*Ganoderma lucidum*	Spore oil	Human lung carcinoma cells LTEP-a2	GLSO significantly decreased VEGF mRNA expression by about 50%.	[41]
*Ganoderma lucidum*	Spore oil/extract spore oil	Human breast cancer cell line MDA-MB-231	GLSO and GLESO significantly decreased VEGF mRNA expression.	[42]
*Ganoderma lucidum*	Spore oil/extract spore oil	Mice implanted with MDA-MB-231	(1) 20 mg/kg, 40 mg/kg GLSO, and 40 mg/kg GLESO significantly reduced tumor weight relative to the control group. (2) GLSO and GLESO significantly decreased VEGF-A mRNA expression.	[42]
*Ganoderma lucidum*	Broken spore powder	Lewis tumor-bearing mice	Broken GLSP significantly reduced tumor weight, volume, and VEGF protein level relative to the normal saline control group.	[43]
*Ganoderma lucidum*	Spore powder	BALB/c mice implanted with HepG2 cells	GLSP significantly reduced tumor volume and VEGF protein level compared to the saline group.	[44]
*Ganoderma lucidum*	Spore oil	Mice implanted with hepatocarcinoma H22 cells	GLSO (7.4 and 14.8 g/kg) significantly reduced tumor volume and VEGF protein level relative to the saline control group.	[45]
*Ganoderma lucidum*	Solaray^®^	Ehrlich Ascites Carcinoma cells-bearing mice	GL significantly reduced tumor volume and VEGF protein level relative to the control group.	[46]
*Coriolus versicolor*	Protein-bound polysaccharides (PBPs)	Murine breast cancer cell line (4T1)	PBP regulated the secretion of various cytokines, including the decreased production of VEGF.	[47]
*Coriolus versicolor*	PSP solution	BALB/c mice implanted with murine sarcoma S180 cells	Compared to the control group, PSP significantly decreased tumor growth, vascular density, and VEGF mRNA expression.	[48]
*Coriolus versicolor*	Polysaccharide (CVE)	Mice implanted with hepatocarcinoma cells HepA	Compared to the control group, CVE significantly decreased tumor weight and VEGF gene expression.	[49]

Abbreviations: CVE, *Coriolus versicolor* polysaccharide extract; GAA, ganoderic acid; GLE, *Ganoderma lucidum* extract; GLPP, *Ganoderma lucidum* polysaccharide peptide; GLPS, *Ganoderma lucidum* polysaccharides; GLSP, *Ganoderma lucidum* spore powder; GLSO, *Ganoderma lucidum* spore oil; GLT, *Ganoderma lucidum* triterpenoids; PSP, polysaccharide peptide.

**Table 3:** Lingzhi/Yunzhi enhanced antitumor activity of EGFR-TKIs in preclinical studies.

Species	EGFR TKI	Chemical Nature of Active Components	Bioactive Compounds/Decoction	Assay/Cells/Model	Major Effects	Reference
*Ganoderma lucidum*	Gefitinib, lapatinib, and sorafenib	-	Extract	HLM	*Ganoderma lucidum* extracts had less effect on the metabolism of the tested anticancers (IC_50_ values >10 μg/mL).	[57]
*Ganoderma colossum*	Gefitinib	Pentacyclic triterpene dilactones	Colossolactone H (colo H) 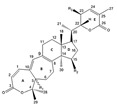	H1650 cell line xenografts in athymic mice (lung cancer)	Combination of colo H and gefitinib had additive cytotoxicity for gefitinib-resistant H1650 lung cancer cells and inhibited the growth of tumor.	[58]
*Ganoderma lucidum*	Lapatinib	-	Extract	Triple-negative IBC SUM149 cell line (breast cancer)	Reishi chemosensitized IBC cells to lapatinib therapy.	[59]
*Ganoderma lucidum*	Erlotinib	-	Extract	IBC cell line, rSUM149 (erlotinib-resistant cells) SCID mice Western blot (breast cancer)	(1) GLE synergized with erlotinib to sensitize SUM-149 cells and overcome erlotinib resistance. (2) Erlotinib/GLE decreased SUM-149 cell viability, proliferation, migration, and invasion. (3) GLE increased erlotinib sensitivity by inactivating AKT and ERK signaling pathways.	[23]
*Ganoderma lucidum*	Sorafenib	Triterpenoid	GL22 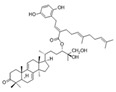	Huh7.5 cells BALB/c nu mice (hepatocellular carcinoma)	(1) GL22 displayed robust antitumor activity against Huh7.5 cells in vitro and in vivo. (2) Combination therapy acts synergistically in the inhibition of HCC.	[60]
*Coriolus versicolor*	Gefitinib	-	XJD *	A549 and H1650 cells xenograft nude mice (NSCLC)	(1) Combination therapy inhibited cell growth, reduced SP1 and EP4 protein expression levels, and HOTAIR levels. (2) Combination treatment resulted in significant reduction in tumor weight and sizes compared to the control group.	[61]

* XJD contain *Psoralea corylifolia* L. (15 g), *Coriolus versicolor* (L. ex Fr.) Quel (15 g), *Astragalus membranaceus* (Fisch.) Bge (30 g), *Curcuma phaeocaulis* Val. (20 g), *Buthus martensii Karsch* (10 g), *Scolopendra subspinipes mutilans* L. Koch (15 g), *Rheum palmatum* L. (10 g), *Hedyotis diffusa* Willd (30 g). HLM: human liver microsome, IBC: inflammatory breast cancer, SCID: severe combined immunodeficient, XJD: Xiaoji decoction, NSCLC: non-small cell lung cancer.

**Table 4:** Effects of Lingzhi on various ABC drug transporters.

Transporter	Species	Nature of the Herbal Materials	Active Component(s) from the Medicinal Mushroom	Concentration	Inhibitor/ Substrate	Other Effect(s)	Reference
P-gp	*Ganoderma lucidum*	extract	-	100 μg/mL	Inhibitor	-	[119]
	*Ganoderma lucidum*	triterpenoid extract	-	100 μg/mL	Inhibitor	-	[119]
	*Ganoderma lucidum*	polysaccharide extract	-	100 μg/mL	No influence	-	[119]
	*Ganoderma lucidum*	triterpene	Ganoderic acid A 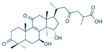	1, 10, 100 μg/mL	No influence	-	[119]
	*Ganoderma lucidum*	polysaccharides	-	10, 50 mg/L	-	Downregulation mRNA expression	[56]
	*Ganoderma lucidum*	triterpene	Ganoderic acid T 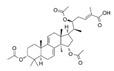	12 μg/mL	Inhibitor	-	[120]
	*Ganoderma lucidum*	triterpene	Ganoderic acid Me 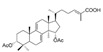	12 μg/mL	Inhibitor	-	[121]
	*Ganoderma lucidum*	ethyl acetate extract	-	20, 100 μg/mL	-	Downregulation protein expression	[122]
	*Ganoderma lucidum*	n-butyl alcohol extract	-	4, 20 μg/mL	-	Downregulation protein expression	[122]
	*Ganoderma lucidum*	triterpene	Ganoderic acids	20 μM	Inhibitor	-	[13]
MDR-1	*Ganoderma lucidum*	ethyl acetate extract	-	20, 100 μg/mL	-	Downregulation gene expression	[122]
	*Ganoderma lucidum*	n-butyl alcohol extract	-	4, 20 μg/mL	-	Downregulation gene expression	[122]
MRP	*Ganoderma lucidum*	extract	-	100 μg/mL	Inhibitor	-	[119]
	*Ganoderma lucidum*	triterpenoid extract	-	100 μg/mL	Inhibitor	-	[119]
	*Ganoderma lucidum*	polysaccharide extract	-	100 μg/mL	No influence	-	[119]
	*Ganoderma lucidum*	triterpene	Ganoderic acid A	1, 10, 100 μg/mL	No influence	-	[119]
	*Ganoderma lucidum*	polysaccharides	-	10, 50 mg/L	-	Downregulation mRNA expression	[56]
BCRP	*Ganoderma lucidum*	extract	-	100 μg/mL	Inducer	-	[119]
	*Ganoderma lucidum*	triterpenoid extract	-	100 μg/mL	Inducer	-	[119]
	*Ganoderma lucidum*	polysaccharide extract	-	100 μg/mL	Inducer	-	[119]
	*Ganoderma lucidum*	triterpene	Ganoderic acid A	1, 10, 100 μg/mL	No influence	-	[119]

**Table 5:** Modulation of CYP isoenzymes by Lingzhi/Yunzhi.

P450 Isoenzyme	Species	Nature of Herbal Materials	Active Component	Model	IC_50_ (µg/mL)	Reference
CYP1A2	*Ganoderma lucidum*	extract	-	HLMs	272.6	[119]
		triterpene		HLMs	364.2	
		polysaccharide		HLMs	*	
		extract		RLMs	*	
		triterpene		RLMs	476.8	
		polysaccharide		RLMs	*	
		triterpene	Ganoderic acid A	HLMs	No inhibition	
		triterpene	Ganoderic acid A	RLMs	No inhibition	
		polysaccharide		CGHM	393	[128]
	*Coriolus versicolor*	polysaccharopeptide	-	HLMs	IC_20_ = 5.4 μM	[129]
CYP2C19	*Ganoderma lucidum*	extract	-	HLMs	131.2	[119]
		triterpene		HLMs	102.5	
		polysaccharide		HLMs	*	
		extract	-	RLMs	261.1	
		triterpene		RLMs	*	
		polysaccharide		RLMs	*	
		triterpene	triterpene	HLMs	No inhibition	
		triterpene	triterpene	RLMs	No inhibition	
CYP2D6	*Ganoderma lucidum*	extract	-	HLMs	164.4	
		triterpene		HLMs	116.1	
		polysaccharide		HLMs	*	
		extract		RLMs	256.8	
		triterpene		RLMs	289.1	
		polysaccharide		RLMs	*	
		triterpene	Ganoderic acid A	HLMs	No inhibition	
		triterpene	Ganoderic acid A	RLMs	No inhibition	
		triterpene	Ganoderic acid A	HLMs	21.83 µM	[130]
	*Coriolus versicolor*	polysaccharopeptide		HLMs	IC_20_ = 15.6 μM	[129]
CYP3A4	*Ganoderma lucidum*	extract		HLMs	150.5	[119]
		triterpene		HLMs	136.4	
		polysaccharide		HLMs	*	
		extract		RLMs	460.8	
		triterpene		RLMs	245.9	
		polysaccharide		RLMs	*	
		triterpene	Ganoderic acid A	HLMs	No inhibition	
		triterpene	Ganoderic acid A	RLMs	No inhibition	
		triterpene	Ganoderic acid A	HLMs	15.05 µM	[130]
	*Coriolus versicolor*	polysaccharopeptide	-	healthy adults	no inhibition or induction	[131]
		polysaccharopeptide	-	HLMs	IC_20_ = 7.6 μM	[129]
CYP2C9	*Ganoderma lucidum*	extract		HLMs	142.2	[119]
		triterpene		HLMs	82.2	
		polysaccharide		HLMs	*	
		extract		RLMs	235.2	
		triterpene		RLMs	163.1	
		polysaccharide		RLMs	*	
		triterpene	Ganoderic acid A	HLMs	No inhibition	
		triterpene	Ganoderic acid A	RLMs	No inhibition	
	*Coriolus versicolor*	polysaccharopeptide		HLMs	IC_20_ = 5.47 μM	[132]
CYP2E1	*Ganoderma lucidum*	extract	-	HLMs	290.2	[119]
		triterpene		HLMs	*	
		polysaccharide		HLMs	305.3	
		extract		RLMs	226.9	
		triterpene		RLMs	387.3	
		polysaccharide		RLMs	*	
		triterpene	Ganoderic acid A	HLMs	No inhibition	
		triterpene	Ganoderic acid A	HLMs	28.35 µM	[130]
		triterpene	Ganoderic acid A	RLMs	No inhibition	[119]
		Polysaccharide	-	CGHM	*	[128]
	*Coriolus versicolor*	polysaccharopeptide	-	HLMs	IC_20_ = 11.9 μM	[129]

*: IC_50_ > 500 µg/mL. HLM: human liver microsome; RLM: rat liver microsome; CGHM: Calmette Guérin (BCG)-stimulated hepatic microsomes.

## Data Availability

Not applicable.
